# A future food boom rescues the negative effects of early-life adversity on adult lifespan in a small mammal

**DOI:** 10.1098/rspb.2023.2681

**Published:** 2024-04-24

**Authors:** Lauren Petrullo, David Delaney, Stan Boutin, Jeffrey E. Lane, Andrew G. McAdam, Ben Dantzer

**Affiliations:** ^1^ Department of Ecology and Evolutionary Biology, University of Arizona, Tucson, 857192, AZ, USA; ^2^ Department of Ecology and Evolutionary Biology, University of Colorado, Boulder, 803023, CO, USA; ^3^ Department of Natural Resource Ecology and Management, Iowa State University, Ames, 500114, IA, USA; ^4^ Department of Biological Sciences, University of Alberta, Edmonton, T6G 2R35, Alberta, Canada; ^5^ Department of Biology, University of Saskatchewan, Saskatoon, S7N 5A26, Saskatchewan, Canada; ^6^ Department of Psychology, University of Michigan, Ann Arbor, 481097, MI, USA; ^7^ Department of Ecology and Evolutionary Biology, University of Michigan, Ann Arbor, 481097, MI, USA

**Keywords:** early-life adversity, mammal, longevity

## Abstract

Early-life adversity, even when transient, can have lasting effects on individual phenotypes and reduce lifespan across species. If these effects can be mitigated by a high-quality later-life environment, then differences in future resources may explain variable resilience to early-life adversity. Using data from over 1000 wild North American red squirrels, we tested the hypothesis that the costs of early-life adversity for adult lifespan could be offset by later-life food abundance. We identified six adversities that reduced juvenile survival in the first year of life, though only one—birth date—had continued independent effects on adult lifespan. We then built a weighted early-life adversity (wELA) index integrating the sum of adversities and their effect sizes. Greater weighted early-life adversity predicted shorter adult lifespans in males and females, but a naturally occurring food boom in the second year of life ameliorated this effect. Experimental food supplementation did not replicate this pattern, despite increasing lifespan, indicating that the buffering effect of a future food boom may hinge on more than an increase in available calories. Our results suggest a non-deterministic role of early-life conditions for later-life phenotype, highlighting the importance of evaluating the consequences of early-life adversity in the context of an animal's entire life course.

## 1. Introduction

In humans, the early environment exhibits such profound predictive power over later-life phenotype that the first 1000 days of life are widely recognized as foundational for determining future health, quality of life and even human capital [1]. Adverse early-life conditions can alter brain development [2], dysregulate immune and endocrine systems [3,4], and increase morbidity and mortality in adulthood [5]. Among non-human animals, a harsh developmental environment can have similar far-reaching effects. Challenging ecological conditions during early life have been associated with higher adult parasite load in rabbits [6], inflammation in birds [7] and poor reproductive performance in hyenas [8]. Most consistently, both ecological and maternal challenges appear to independently and additively predict reduced adult lifespan across taxa [9–12]. Juvenile animals can struggle to access maternal resources due to poor maternal condition, mistimed parturition or sibling competition [13–16]. The potential mechanisms linking early-life challenges to reduced lifespan include physiological damage to telomeres [17–19], life-history trade-offs that deprioritize the developmental systems that promote longevity [15,20], and accelerated reproductive development at the expense of longevity ([21–23], but see [10]).

Sex-specific susceptibility to early-life adversity has been demonstrated in some species, with negative associations between harsh developmental conditions and later-life phenotypes apparent in only one sex [24–28]. When longevity differs between the sexes (e.g. female mammals typically outlive males, [29]), sex-specific selection pressures may result in differential sensitivity to the environmental challenges that can reduce lifespan. Moreover, because individuals can be exposed to many forms of early-life adversity simultaneously, these challenges can combine to collectively reduce longevity [8,30], and their unique combinations may have synergistic effects [31]. This has fuelled an interest in understanding how animals inhabiting heterogeneous environments may cope with clusters of adversity during development, and whether one sex is more susceptible to the consequences of cumulative adversity. Identifying these effects can also provide insight into how animals will respond to multidimensional environmental shifts caused by human-induced rapid environmental change (HIREC), which can generate distinct but co-occurring sources of early-life adversity [32].

Notably, emerging work suggests that the quality of an individual's future environment may buffer against, or magnify, the costs of harsh early-life environments. In non-human primates, an individual's social environment predicts longevity [33], and strong social bonds and high social status during adulthood can ameliorate the negative effects of early-life adversity on survival [34]. Beyond sociality, future food availability may similarly modify the long-term effects of early-life adversity, particularly in less social species. In experimental models, a food-scarce environment in adulthood can exacerbate the consequences of early-life adversity for adult lifespan [35]. In nature, animals inhabiting resource pulse ecosystems can experience pronounced temporal variation in food availability and thus dramatic within-generation fluctuations in environmental quality: individuals can be born into a food-scarce environment but subsequently experience a food boom in the future [36]. Food booms act as natural experiments that temporarily increase environmental quality, potentially freeing individuals from the constraints of early-life adversity by increasing available calories and/or by driving population-level dynamics (e.g. reducing competition). If the effects of early-life adversity can be modified by later-life environmental quality, variation in future conditions may explain variation in resilience to the negative consequences of a harsh developmental environment.

In this study, we tested the overarching hypothesis that future environmental quality modifies the relationship between early-life adversity and adult lifespan. We leveraged 32 years of data on 1144 wild North American red squirrels (*Tamiasciurus hudsonicus*, *N* = 885 control squirrels and *N* = 259 experimental squirrels) inhabiting a resource pulse ecosystem in Yukon, Canada. In this population, natal dispersal distances are relatively small in both sexes (102 metres ± 107 s.d., [37]), and adult lifespan is positively associated with lifetime reproductive success [38]. Masting by white spruce (*Picea glauca)*, the preferred food source of red squirrels, causes dramatic interannual variability in food availability through food booms (mast years) and busts (non-mast years) [39]. These fluctuations, combined with variable weather, predator abundance, intraspecific competition and maternal investment, can create exceptionally harsh developmental conditions for some squirrels (e.g. annual juvenile mortality ranges from 6 to 70%, [40]). Thus, we first tested each measure of the early environment as a putative source of early-life adversity to identify those that significantly reduced the probability of juvenile survival into adulthood (table 1*a*).

**Table 1.** (*a*) Hypothesized sources of early-life adversity in juvenile squirrels experienced during their first year of life and (*b*) potential buffers against early-life adversity based on prior work involving red squirrels.

variable	definition	references
**a) potential sources of early-life adversity**		
*large litter size*	relatively large number of siblings born within the same litter (increased sibling competition for maternal resources and conspecific competition for territories)	[48]
*later birth date*	date of birth is relatively late in the breeding season (increased conspecific competition for territories)	[40,72]
*slow postnatal growth*	relatively low offspring growth rate during period of maternal dependence calculated as change in body mass from approximately 0 days to 25 days of age (reduced likelihood of territory acquisition)	[40,44,59]
*non-mast year*	a year in which a white spruce mast does not occur (food scarcity)	[73]
*elevated squirrel density*	relatively high number of squirrels living on study area (increased conspecific competition)	[42,43,59,74]
*1 year following peak in lynx-hare cycle*	a year characterized by a crash in hare density and expected prey-switching by lynx to red squirrels (increased likelihood of predation)	[46]
*elevated mustelid density*	relatively high number of nest predators (increased likelihood of predation)	[42,64]
*mean winter temperature*	relatively cold average winter temperatures (increased thermoregulatory costs)	[42,48]
*large litter x non-mast year*	litter size mismatched to future food abundance (i.e. a large litter produced in a non-mast year when future food is low)	[43,44]
*elevated squirrel density x later birth date*	relatively high number of squirrels living on study area and a later birth date (reduced likelihood of territory acquisition)	[40,59,72]
*elevated squirrel density x slow postnatal growth*	relatively high number of squirrels living on study area and poorer growth (reduced likelihood of territory acquisition)	[59]
**b) potential buffers against early-life adversity**		
*mast year*	encountering a spruce mast event, which results in the production of a superabundance of food, during the second year of life	[73]
*experimental food supplementation*	access to a supplemental feeding station at the centre of a squirrel's territory	[75]

Cumulative sum measures of early-life adversity may be especially useful for revealing additive consequences of concurrent environmental challenges [30,41]. Although some studies find multivariate models of adversity to outperform such measures [8], it remains unclear how a cumulative measure accounting for the relative effect sizes of different sources of adversities performs in comparison. We therefore built an integrative, weighted early-life adversity (wELA) index to quantify the early-life environments of juveniles. We hypothesized that wELA could influence longevity in two ways: first, by reducing adult lifespan through detrimental carry-over effects on phenotypic quality (i.e. a negative relationship), and second, by increasing the phenotypic quality of survivors through the selective disappearance of poor-quality individuals from the population early in life (i.e. a positive relationship). We then tested whether the consequences of early-life adversity on lifespan could be ameliorated by a high-quality future environment using two measures of food abundance: a naturally occurring, population-wide food boom occurring in the second year of life, and experimental supplementation with an additional food source (table 1*b*). We predicted that in both cases, future food abundance would offset, at least in part, any negative effects of wELA in the first year of life on adult lifespan.

## 2. Results

### (a) Early-life adversity predicts variation in juvenile survival and adult lifespan

**Figure 1.**
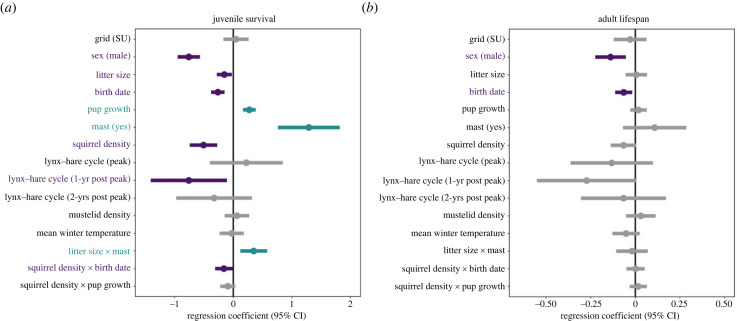
Harsh conditions in the first year of life independently predict poor juvenile survival and reduced adult lifespan. (*a*) Six of the eight potential early-life adversities, and their interactions, were associated with a reduced likelihood of juvenile overwinter survival (i.e. survival past the first 200 days of life). (*b*) Birth date was the only factor to demonstrate a continued effect on adult lifespan for those individuals that survived their first winter. Forest plots reflect results of generalized linear mixed-effects models (*a*: binomial, *b*: Poisson) testing which early-life factors predict juvenile overwinter survival (*N* = 3699 squirrels) and total adult lifespan (*N* = 885 squirrels). Purple bars denote factors that significantly (*p* < 0.05) negatively correlate with survival; green bars denote factors that significantly positively correlate with survival; grey bars denote non-significant factors. SU, study area/grid named Sulphur.

Following variable standardization, six of the eight variables tested, and their interactions, were significantly associated with juvenile survival (survival over the first winter, i.e. > 200 days) in the first year and thus defined as early-life adversities (figure 1*a*, table 2*a*). The strongest predictor of juvenile survival was whether it was a spruce mast (food boom) year or not, followed sequentially by year in the lynx-hare cycle, squirrel density, postnatal growth rate, birth date and litter size. Juveniles were less likely to survive their first winter if they were born in non-mast years when food was scarce (*β* = 1.29, *z* = 4.79, *p* < 0.0001) or in the year following the peak of the lynx-hare cycle when there was a crash in the snowshoe hare population and lynx prey-switch to squirrels (*β* = −0.76, *z* = −2.28, *p* = 0.022). Juveniles were also less likely to survive when squirrel densities were elevated (*β* = −0.51, *z* = −4.25, *p* < 0.0001), and if they grew slowly during the early postnatal period of maternal dependence (i.e. first 25 days; *β* = 0.27, *z* = 4.81, *p* < 0.0001), were born later in the breeding season (*β* = −0.27, *z* = −4.54, *p* < 0.0001; particularly if conspecific density was also high, *β* = −0.16, *z* = −2.11, *p* = 0.035), or were born into a large litter (*β* = −0.15, *z* = −2.32, *p* = 0.020) or a litter whose size was mismatched to food production that year (e.g. a large litter in a non-mast year, *β* = 0.35, *z* = 2.968, *p* = 0.003). There was no effect of mean overwinter temperature or the abundance of nest predators (mustelids) on juvenile survival. Of the factors identified as early-life adversities, only birth date exhibited continued, independent effects on adult lifespan beyond juvenile survival such that squirrels that were born later in the breeding season lived shorter adult lives than those born earlier (*β* = −0.07, *z* = −2.70, *p* = 0.007, figure 1*b*, table 2*b*). Males were less likely to survive their first winter compared with females (*β* = −0.76, *z* = −7.83, *p* < 0.0001, figure 1*a*, table 2*a*), and when they did, they lived shorter adult lives (*β* = −0.14, *z* = −3.18, *p* = 0.001, figure 1*b*, table 2*b*).

**Table 2.** Harsh early-life conditions predict poorer juvenile survival but have limited independent effects on total adult lifespan. Results show output from generalized linear mixed-effects models (binomial and Poisson). Continuous predictors were centred to a mean of zero and expressed in standard deviations. Model 'a' is binomial, model 'b' is Poisson. Bold values indicate significant (*p* < 0.05) variables.

independent terms	(a) juvenile survival (*N* = 3699)				(b) adult lifespan (*N* = 885)			
	estimate	s.e.	Z	*p*	estimate	s.e.	Z	*p*
intercept	**−1.31**	**0.16**	**−8.30**	**< 0.0001**	**1.03**	**0.06**	**17.36**	**< 0.0001**
grid (SU)	0.05	0.11	0.43	0.670	−0.03	0.05	−0.62	0.533
**sex (male)**	**−0.76**	**0.10**	**−7.83**	**< 0.0001**	**−0.14**	**0.04**	**−3.18**	**0.001**
**litter size**	**−0.15**	**0.07**	**−2.32**	**0.020**	0.01	0.03	0.17	0.865
**birth date**	**−0.27**	**0.06**	**−4.54**	**< 0.0001**	**−0.07**	**0.02**	**−2.70**	**0.007**
**growth rate**	**0.27**	**0.06**	**4.81**	**< 0.0001**	0.02	0.02	0.71	0.477
**mast (yes)**	**1.29**	**0.27**	**4.79**	**< 0.0001**	0.11	0.09	1.20	0.231
**squirrel density**	**−0.51**	**0.12**	**−4.25**	**< 0.0001**	−0.07	0.04	−1.81	0.071
lynx-hare cycle (peak)	0.22	0.32	0.70	0.482	−0.13	0.12	−1.12	0.262
**lynx-hare cycle (1-yr post peak)**	**−0.76**	**0.33**	**−2.28**	**0.022**	−0.27	0.14	−1.91	0.056
lynx-hare cycle (2-yrs post peak)	−0.33	0.33	−0.99	0.321	−0.07	0.12	−0.55	0.585
mustelid density	0.06	0.11	0.58	0.559	0.03	0.04	0.72	0.473
mean winter temperature	−0.03	0.10	−0.25	0.800	−0.05	0.04	−1.33	0.183
**litter size x mast (yes)**	**0.35**	**0.12**	**2.98**	**0.003**	−0.02	0.05	−0.42	0.675
**birth date x squirrel density**	**−0.16**	**0.08**	**−2.11**	**0.035**	0.001	0.03	0.05	0.961
growth rate x squirrel density	−0.09	0.07	−1.43	0.153	0.02	0.02	0.67	0.503
**random effects**				**variance**				**variance**
litter identity				0.76				0.01
maternal identity				0.00				0.02
cohort				0.21				0.02

### (b) A naturally occurring food boom mitigates the longevity costs of early-life adversity

For each squirrel that survived its first winter to the following spring, we generated a wELA index that integrated the number of adversities experienced in their first year of life, as well as their relative effect sizes (electronic supplementary material, figure S1). wELA explained a substantial amount of variation in adult lifespan, outperforming other measures of early-life adversity (e.g. a count/sum index and birth date only, electronic supplementary material, table S1). Overall, individuals with greater wELA indices died sooner than those with smaller indices (*β* = −0.12, *z* = −3.41, *p* = 0.0006, electronic supplementary material, table S2). Squirrels with wELA indices at the third quartile compared with the first suffered a predicted 14% decrease in adult lifespan (from 2.97 to 2.56 years). We found no evidence for sex-specific effects of wELA on lifespan as the negative relationship between wELA and longevity did not significantly differ between males and females (electronic supplementary material, table S2).

**Figure 2.**
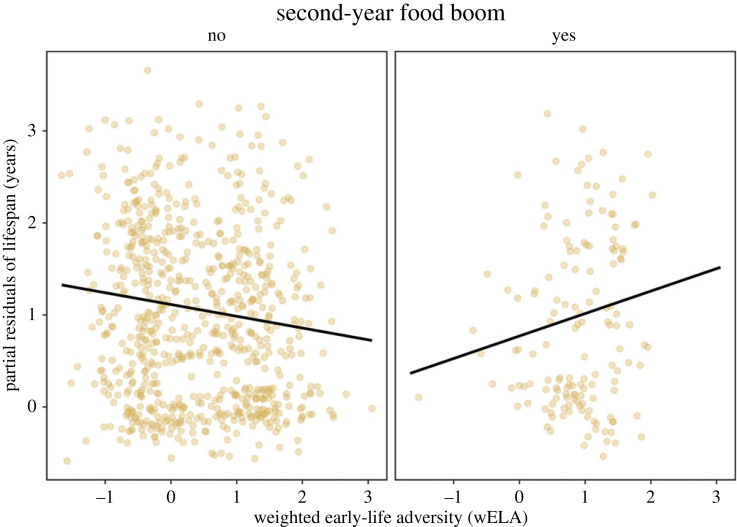
A second-year food boom mitigates the costs of early-life adversity for adult lifespan. Squirrels that experienced harsher conditions in their first year of life lived shorter lives (electronic supplementary material, table S2). For squirrels that did not experience a food boom event (i.e. spruce mast) in their second year of life, greater weighted early-life adversity (wELA) led to steeper declines in adult lifespan. For squirrels that did encounter a second-year food boom, greater wELA was instead associated with increased adult lifespan. Plot depicts partial residuals from a generalized linear mixed-effects model (Poisson; *N* = 885 squirrels).

White spruce masts are food boom events characterized by the episodic production (every 3–7 years) of a superabundance of food that can be accessed by the entire population of red squirrels at our study site. Greater wELA was associated with a decrease in adult lifespan for individuals that suffered food scarcity in their second year of life, but for those that encountered a second-year food boom, the effects of wELA for adult lifespan were reversed (electronic supplementary material, table S2). Greater wELA predicted greater adult longevity among squirrels that experienced an increase in future environmental quality (*β* = 0.33, *z* = 3.07, *p* = 0.002; figure 2, electronic supplementary material, table S2).

### (c) Experimental supplementation does not recapitulate these patterns

**Figure 3.**
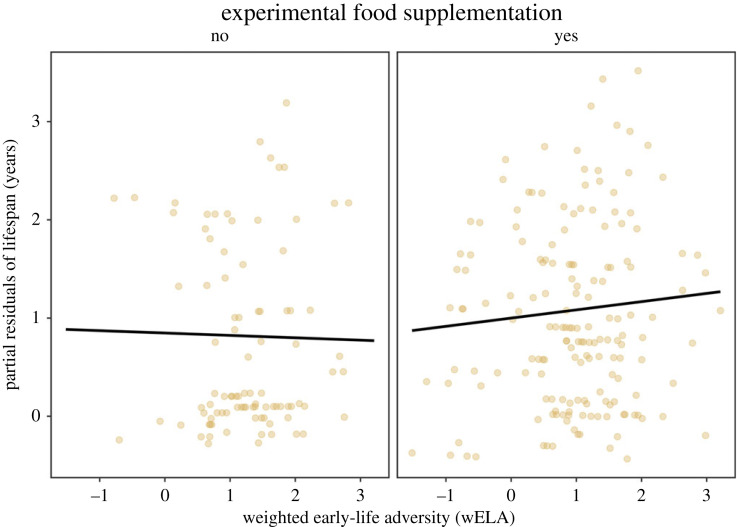
Experimental food supplementation extends lifespan but does not alter the relationship between early-life adversity and longevity. On the experimental study area, squirrels that received peanut butter at the centre of their territories lived longer adult lives than those that did not receive a bucket (electronic supplementary material, table S3), but supplemental food did not modify the relationship between wELA and adult lifespan. Plot depicts partial residuals from a generalized linear-mixed effects model (Poisson; N = 259 squirrels).

We experimentally supplemented a subset of individuals with an additional food source in an attempt to replicate the buffering effect of a food boom against the negative consequences of early-life adversity. On a separate study area, we provided some squirrels with a bucket containing 1 kg of peanut butter at the centre of their individual territories, accessible ad libitum to the supplemented individual throughout the course of their lives. Squirrels that received peanut butter outlived those that inhabited the same study area but did not receive peanut butter (*β* = 0.27, *z* = 3.01, *p* = 0.003; figure 3, electronic supplementary material, table S3). However, food supplementation had no modifying effect on the relationship between wELA and lifespan, which was non-significant for all squirrels in the experimental study area (electronic supplementary material, table S3).

## 3. Discussion

The first winter of a young Yukon red squirrel's life is the most crucial survival bottleneck it will experience [38,42]. The strongest predictors of juvenile mortality during this time–and thus, the most influential forms of early-life adversity–were ecological factors related to food, predation, and competition. As we have previously shown, squirrels born in non-mast years when new food production was scarce were less likely to survive than pups born in the months ahead of an autumn food boom (mast years) [43]. This was especially true when maternal reproductive output was mismatched to the food availability a squirrel would experience during the periods of weaning and independence [44]. For instance, juveniles were less likely to survive if they were born into a large litter in a non-mast year, likely due to increased competition with a greater number of siblings for both food and territories. Elevated squirrel densities (and thus competition) more broadly also reduced the likelihood of juvenile survival, as did relevant predator-prey cycles. Canada lynx exhibit prey-switching from snowshoe hares to red squirrels in the year after hare populations crash, so squirrels born in the year following a hare crash are at the highest risk of succumbing to, and witnessing, predation events [45,46], and thus less likely to survive into adulthood. Juvenile survival was also lower among squirrels likely to encounter greater sibling competition and curtailed maternal investment. Sibling competition for maternal resources is greatest in large litters and manifests as a quantity versus quality trade-off in which pups born into larger litters suffer slower growth [47], which reduces a squirrel's ability to compete for its own natal territory as well as territories adjacent to it [48].

For squirrels born later in the year, the likelihood of encountering vacant territories is low given that most territories will already be occupied by earlier-born squirrels. Territory acquisition is critical for overwinter survival [42], thus later-born squirrels face increased conspecific competition for territories and a consequent increase in mortality risk [48]. But unlike recent work in Colombian ground squirrels demonstrating no carry-over effects of birth date on adult longevity [49], we found birth date to be the only early-life factor that exhibited continued, independent effects on adult lifespan. Later-born squirrels that survived their first winter exhibited shortened adult lifespans compared with earlier born squirrels. For these individuals, the energetic demands of navigating nutritional and spatial independence in an increasingly competitive environment may accelerate phenotypic deterioration through the shortening of telomeres [17–19], or may shift resource allocation decisions, either of which may manifest as a reduction in adult lifespan.

Environmental covariance can generate clusters of adversity in which multiple ecological challenges co-occur [50]. The consequences of cumulative adversity may therefore be particularly profound in populations inhabiting unstable or rapidly shifting environments [8,30], and may depend on the relative magnitudes of different types of adversity. We hypothesized that wELA (the number of early-life adversities experienced and their relative effect sizes) could reduce adult lifespan through physiologic damage (e.g. ‘silver-spoon’ hypothesis, [51]), or increase adult lifespan through viability selection (e.g. selective disappearance of poor-quality individuals). We found support for the former hypothesis: individuals with greater wELA exhibited reduced adult lifespan, and later-born squirrels exhibited both poorer juvenile survival and reduced lifespan, suggesting detrimental carry-over effects. If selection had removed poorer quality individuals from the population, wELA and/or later birth dates may have instead predicted longer adult lives. It is possible, however, that both mechanisms, which likely exert opposing effects, acted simultaneously but that detrimental carry-over effects outweighed the effects of selective disappearance.

Unlike previous studies in other mammals (e.g. [24,27,52]), we found no evidence for sex differences in the relationship between wELA and adult lifespan. Although males were less likely to survive their first winter and had shorter adult lifespans than females when they did survive, both sexes exhibited reduced longevity associated with cumulative exposure to harsh environmental conditions in early life. This lack of sex specificity in environmental sensitivity may be a result of sexual monomorphism in body size in red squirrels [53], and thus similar energetic/nutritional requirements and vulnerability to environmentally induced energetic constraints [54]. Selection pressures during the juvenile period may also be similar in both sexes, reflecting parallel fitness costs of life-history plasticity. Indeed, prior work in our study population has failed to find evidence of sex specificity in selection for postnatal growth rate or birth date beyond the lowest level (i.e. within litter) [55].

Human research has long endeavoured to explain how the biological embedding of early-life adversity leads to variation in individual health and longevity [56,57]. What remains largely unknown are what, if any, factors can buffer against such embedding. If the negative effects of early-life adversity are non-deterministic such that they can be mitigated by future conditions, then consideration of an individual's entire life course is essential to explaining variation in susceptibility to developmental challenges. We tested for a mitigation effect of food booms that occurred in the second year of life, which could theoretically modify the longevity costs of early-life adversity without altering the strength of past selection during the first year of life. Contrary to the predictive adaptive response (PAR) hypothesis, which predicts that unfavourable developmental conditions can prime individuals for adaptive responses to similarly unfavourable adult conditions [58], individuals with greater wELA during development still suffered longevity costs if the future environment was similarly challenging (i.e. food scarce). Instead, individuals with greater wELA that experienced a second-year food boom lived longer adult lives than those with lower wELA in spite of harsh developmental conditions.

We have previously shown that, although they occur episodically, the boom of food produced in mast years enhances absolute fitness in red squirrels, increasing both annual and lifetime reproductive success [43,44]. Here, our results suggest that food booms can also alleviate the longevity costs of harsh early-life conditions, perhaps by magnifying survivorship bias resulting from developmental challenges. Individuals resilient enough to overcome greater wELA may have had a ‘phenotypic edge’ that allowed them to maximize the longevity benefits of a food boom. Indeed, among squirrels encountering a second-year food boom, those with greater wELA outlived those with lower wELA. As some degree of environmental autocorrelation is unavoidable in this system (e.g. those experiencing a mast in their second year of life would not have been born during a mast and *vice versa*), experimental work is necessary to parse alternative developmental drivers of variation in adult lifespan. Nonetheless, masting by white spruce appears to serve both as a principal force of early-life adversity (e.g. when squirrels are born into food-scarce conditions), as well as a potential buffer against the longevity costs of a harsh developmental environment.

We were unable to replicate these buffering effects by providing squirrels with ad libitum peanut butter, despite our prior work showing that supplementation can ameliorate other constraints like the trade-off between litter size and pup growth [59], and the fitness costs of having large litters in low food years [44]. Experimental supplementation did increase adult lifespan overall, suggesting that food availability may constrain adult lifespan in our study population. However, there was no relationship between wELA and adult lifespan on the experimental study area, and no modifying effect of food supplementation. Spruce masting is inherently different from experimental supplementation: it is a predictable and episodic environmental change with which squirrels in this region have co-evolved, and which alters behaviour, fitness, life histories and population dynamics at a large scale [43,44,60,61]. We experimentally replicated only one component of a food boom (e.g. increased available calories), and it was smaller in scale, individualized, and continuous. Food supplementation may therefore not elicit a buffering effect if that effect depends on an alternate component of spruce masting (e.g. we have also been unable to replicate the increase in litter size and number induced by cues of an upcoming mast event using peanut butter, [44]). Further, because supplementation substantially increased adult lifespan (*β* = 0.27), it may have reduced the variation in longevity needed to capture susceptibility to harsh developmental conditions. Supplemented squirrels on this study area may thus be resilient to the longevity costs of early-life adversity because their adult lifespan has been extended beyond the range of sensitivity to challenging environmental conditions in the first year of life.

In sum, our findings extend our current understanding of the scope of early-life effects in wild animals. We show that early-life challenges can have cumulative, additive consequences for adult lifespan in both sum and relative magnitude, highlighting the benefits of using a weighted measure of early-life adversity to capture synergistic effects of different developmental variables [31]. We uncovered one dimension of the future environment, population-wide food availability, that can buffer against the negative effects of early-life adversity. Although food booms and their effects may be unique to resource pulse systems, they reflect pronounced, transient increases in environmental quality, and can serve as natural experiments that mimic large-scale environmental perturbations [36]. Our findings therefore provide evidence that interindividual variation in vulnerability and resilience to the long-term consequences of early-life adversity may hinge on broader patterns of competition and constraint at the population level.

## 4. Material and methods

### (a) Study system

All data for the current study were collected from 1989 to 2022. During this time, we continuously monitored North American red squirrels (*Tamiasciurus hudsonicus*) in the southwest Yukon, Canada (61°N, 138°W) [39]. Detailed information about the study system and field methods can be found elsewhere [38,39]. Briefly, we followed squirrels from birth until death on two separate approximately 40 hectare study areas (Kloo or ‘KL’ and Sulphur or ‘SU’) as well as on an experimental study area (Agnes or ‘AG’). We identified individual squirrels using uniquely labelled metal ear tags placed shortly after birth while still in their natal nest or at first capture during regular live-trapping. We censused the entire population in May and August/September of each year. Because red squirrels are both territorial and highly trappable, we are able to estimate death and lifespan with high confidence (median adult lifespan = 3.5 years, maximum adult lifespan = 9 years; [38,62]). Male and female red squirrels have similar natal dispersal distances, recruiting an average of two territory widths away from their natal territories [63].

### (b) Life-history and fitness data

We determined female reproductive status via abdominal palpation for fetal development and by monitoring individual mass gain during regular live-trapping. Within a few days of birth, we located each nest using radio-telemetry, counted, ear clipped (for unique marking within each litter and tissue sample) and weighed each pup (to the nearest tenth of a gram). About 25 days later, we reweighed each pup and affixed a set of permanent metal ear tags. Because growth is linear during this period of development [40], we calculated pup postnatal growth rate as the average mass gain per day.

### (c) Temperature data

We used daily temperature records from the Haines Junction weather station, which is located approximately 35 km southeast (SE) from our study area (Climate ID 2100630, 60.77°N, 137.57°W), to calculate yearly mean overwinter temperatures from the months of October to the following March. Prior studies in our population using data from this weather station demonstrate that mean overwinter temperatures capture thermoregulatory costs of temperature extremes and influence juvenile survival and litter failure [42,64]. Study areas were located within 5 kilometres of each other, experiencing similar thermal conditions.

### (d) Predator data

We used data on two types of predators of juvenile red squirrels from our study area, Canada lynx (*Lynx canadensis)* and mustelids (short-tailed weasel *Mustela erminea,* least weasel *M. nivalis* and marten *Martes americana*), from the Kluane Boreal Forest Ecosystem Project (1987–1996) and the Community Ecological Monitoring Program (1996–present). Lynx and mustelid densities (all three mustelid species grouped together) were calculated as the average snow track count per 100 kilometre transect [65]. We also calculated the density of snowshoe hares (*Lepus americanus)*, on which Canada lynx specialize, using mark-recapture [65] because lynx prey-switch to red squirrels following crashes in hare population densities [45]. Following prior studies [46], we binned the hare-lynx cycle into four categories based on the location in the cycle: peak hare density (when both hare and lynx density are high), 1 year post-hare peak (when hare density crashes, but lynx density remains high), 2 years post-peak (when lynx density crashes) and any other year in the cycle. Low juvenile survival suggests red squirrel predation risk from lynx is highest 1 year post-hare peak across study areas [46].

### (e) Measures of food availability

#### (i) Food abundance

Red squirrels in this region primarily consume seed from masting white spruce (*Picea glauca)* trees, which produce a superabundance of food in the autumn (mid-August) episodically every 3–7 years during food boom events, and little to no food in the years between [39]. Over the 32 years of the current study, six separate spruce mast (food boom) events occurred (cone distributions described in previous studies, [39,46]). In all years (mast and non-mast), we counted the number of visible cones on one side of the top third of a consistent subset of trees (between 159 and 254 trees) on each study area [66]. We then log (+1) transformed counts and calculated the mean to represent an annual index [60]. We defined years with a superabundance of cones as mast years, which occur once every 3–7 years [43].

#### (ii) Experimental food supplementation

From 2005 to 2017 except for years following the 2010 and 2014 masts, we experimentally supplemented a subset of squirrels living on a separate study area (AG) by hanging a bucket containing 1 kg of peanut butter between two trees at the centre of a subset of squirrels' territories. Thus, a subset of squirrels on this study area received additional food (supplemented) while others did not (controls). We have previously estimated that 1 kg of peanut butter can exclusively support a squirrel's basal metabolic rate for approximately 70 days [67,68]**.** We replenished peanut butter for supplemented squirrels approximately every 6 weeks from October to May. Given the high territoriality and rarity of pilfering in our study population [69,70], we did not expect that control squirrels had access to the buckets belonging to supplemented squirrels. We have previously found that squirrel density on the food supplemented study area was 65% higher than control areas [39], food supplemented females had increased antioxidant protection and reduced plasma protein oxidative damage compared with unsupplemented females [67], and natural food stores were larger on the food-supplemented study area compared with control areas [69].

#### (iii) Covariance among life-history traits and environmental variables

We standardized life-history traits (birth date, growth rate and litter size) within years, so that values would be relative to the annual mean to remove any covariation between life-history traits and annual environmental variables. Among continuous predictors, absolute values of correlations were ≤ 0.25 (electronic supplementary material, figure S2), with some covariation among discrete and continuous environmental variables (electronic supplementary material, figure S3). Two masts occurred 2 years after a peak in the lynx-hare cycle and no masts co-occurred with a peak or 1 year following a peak in the lynx-hare cycle (see figure 2 in [46]).

### (f) Statistical analysis

We conducted all analyses in R version 4.0.2 (R. Core Team 2015). We used the package *lme4* to conduct generalized linear mixed models (GLMM) and the package *visreg* to visualize partial regressions. We controlled for study area (fixed effect), litter identity (random effect) and year/cohort (random effect) in all models. Some models also contained mother identity as an additional random effect if the model would converge with this additional structure.

#### (i) Defining early-life adversity

We defined early-life factors as adversities experienced in the first year of life that significantly reduced the likelihood of juvenile survival from eight putative adversities based on prior work in our study population (table 1). We defined juvenile survival as surviving to 200 days old and being recorded in our May census, which reflects overwinter survival to the subsequent breeding season regardless of variation in birth date [40,63,71]. To do this, we constructed a model to test which of these factors experienced during a squirrel's birth year were related to survival over a squirrel's first winter to the following May (i.e. spring census) using a binary error distribution (survived yes/no). We used pup growth rate, litter size (mean ± s.d.= 3.6 ± 1 pups, range: 1–8), birth date (day-of-year), mast year (yes/no), year in the hare-lynx cycle (hare peak, 1 year post-peak, 2 years post-peak, other), squirrel population density, mustelid density and mean winter temperature (see rationale for these predictions in table 1). We also included interactions of litter size x mast, birth date x squirrel density, and pup growth rate x squirrel density as predictors as the effects of these factors on juvenile survival may be dependent on other co-occurring variables (e.g. being born late in the year may only have a negative effect if conspecific competition that year is high). To determine if early-life adversities exerted continued, independent effects on lifespan beyond the juvenile period, we ran an identical model to the one described above except we used adult lifespan (i.e. longevity conditional upon survival to 200 days) as the dependent variable rather than juvenile survival. We used a Poisson error distribution with a log link function for all models with adult lifespan as the response.

#### (ii) Construction of a weighted cumulative early-life adversity (wELA) index

We then tested whether early-life adversities exhibited cumulative effects on lifespan. To do this, we created a cumulative, wELA that incorporated both the number and magnitude of adversities an individual experienced, as well as their continuous values. For each significant predictor of juvenile overwinter survival, we extracted the regression coefficient (multiplied by −1 to indicate that positive values represented increased adversity) and multiplied each with the value of the environment or life-history trait for each squirrel. For significant interactions, we multiplied the value of the environment experienced with each individual's life-history trait value and the interaction coefficient. We then summed the strength of each axis of the environment to represent a cumulative index of early-life adversity.

#### (iii) Effects of a future food boom on the relationship between early-life adversity and adult lifespan

We built a GLMM (Poisson) examining the relationship between the wELA index (fixed effect) and lifespan (response), including an interaction between wELA and whether an individual encountered a spruce mast during its second year of life (binary variable yes/no). We did not consider interactions of early-life adversity with spruce masts encountered in the third or later years of life due to reduced sample size and an increasingly shrinking distribution of lifespans. We confirmed this effect was linear by additionally testing for quadratic (*z* = 0.2, *p* = 0.849) and cubic (*z* = −1.1, *p* = 0.260) terms and fitting splines with general additive models.

#### (iv) Food supplementation experiment

We tested whether providing animals with a supplemental food source could ameliorate the cost of early-life adversities on lifespan. We restricted this analysis to cohorts born between 2004 and 2015 to focus on cohorts that had the potential to live at least 4 years before monitoring ended on the experimental study area in 2019. In this dataset, squirrels (*N* = 259 individuals, 10 cohorts) either received a bucket of peanut butter on their territory or did not (binary variable). We ran the same GLMM as above except we included whether an individual received a bucket or not (binary variable yes/no) in place of a spruce mast in the interaction with wELA.

## Data Availability

All data and code used in this project are available at Figshare: https://doi.org/10.6084/m9.figshare.23660442 [76]. Supplementary material is available online [77].
